# Gender differences in the bidirectional relationship between alcohol consumption and sleeplessness: the Tromsø study

**DOI:** 10.1186/s12889-019-6801-6

**Published:** 2019-04-29

**Authors:** Kamilla Rognmo, Svein Bergvik, Jan Harald Rosenvinge, Katja Lovise Bratlid, Oddgeir Friborg

**Affiliations:** Department of Psychology, UiT the Arctic University of Norway, P.O. Box 6050 Langnes, 9037 Tromsø, Norway

**Keywords:** Alcohol use, Sleeplessness, Population sample, Bidirectional study, Longitudinal study

## Abstract

**Background:**

The degree to which the relationship between alcohol use and sleeplessness is unidirectional or reciprocal is unclear due to great variation among the results of previous studies. The aim of the present study was to investigate if the relationship between alcohol use and sleeplessness is bidirectional by exploring how the change in and stability of alcohol use were related to sleeplessness, and vice versa, how the change in and stability of sleeplessness were related to alcohol use, in a longitudinal study spanning 13 years.

**Method:**

Data were collected from 9941 adults who participated in two waves (T1: 1994–1995, and T2: 2007–2008) of the Tromsø Study, a Norwegian general population health study. Alcohol use was measured by questions asking about the frequency of drinking, amounts of alcohol normally consumed and the frequency of binge drinking, whereas sleeplessness was measured by one item asking about the frequency of experiencing sleeplessness. Variables representing change in and stability of consumption of alcohol and sleeplessness from T1 to T2 were created. Logistic regression analyses, stratified by gender, were used to analyze the data.

**Results:**

Men reporting stable high (*OR* = 2.11, *p.* < .001) or increasing (*OR* = 1.94, *p.* < .01) consumption of alcohol from T1 to T2 had a significantly higher risk of reporting sleeplessness at T2. Likewise, men experiencing stable (*OR* = 1.84, *p.* < .01) or increasing (*OR* = 1.78, *p.* < .001) sleeplessness from T1 to T2 had a significantly higher risk of reporting high consumption of alcohol at T2. No significant effects were detected among women.

**Conclusion:**

The findings indicate a bidirectional relationship between high consumption of alcohol and sleeplessness only among men. Thus, healthcare professionals ought to be informed about the health risks associated with excessive drinking and struggling with sleeplessness, especially in men.

The study of alcohol use and sleep has a long history [1]. Drinking alcohol immediately affects sleep architecture and sleep quality [2], such as by suppressing REM sleep and increasing slow-wave sleep during the first half of the sleep period [1]. In addition, drinking alcohol impacts neuroendocrine and neurobiological responses related to sleep, such as GABA facilitation and inhibition of the excitatory activity of the NMDA receptor [3, 4]. Alcohol use and sleep problems are also longitudinally related, as insomnia is more prevalent among people struggling with alcohol use disorders, with prevalence rates ranging between 36 and 91%, compared to prevalence rates between 10 and 15% in the general population [5]. Despite the evident relationship, the influence of alcohol use on the development of sleep problems or vice versa is uncertain.

The findings from population-based studies examining longitudinal relationships between sleep problems, such as insomnia, and alcohol use portray a mixed picture. Most studies find that insomnia predicts alcohol use or abuse [6–8]. Interestingly, longitudinal studies examining the opposite direction tend to find that alcohol use is not a significant predictor of insomnia [9–13]. One population-based study [14] did, however, observe a doubled risk of insomnia, as measured 13 years later, for individuals diagnosed as alcohol dependent. Remission in alcohol dependency was unrelated to the risk of insomnia, but this was true only for those who continued to drink while in remission. Abstinent individuals in remission had an increased risk of insomnia 13 years later. This may suggest that there are other factors related to alcohol-use disorders that enhance the risk of insomnia, such as the severity or chronicity of the disorder.

To our knowledge, only one study has investigated the bidirectionality of this relationship among adults in the general population. Haario and colleagues [15] found that frequent symptoms of insomnia predicted subsequent heavy drinking and vice versa, heavy drinking significantly predicted subsequent symptoms of insomnia.

The aim of the present study was to investigate the directionality of the relationship between alcohol use and sleeplessness as a core symptom of insomnia [16]. In particular, we examined whether change in and stability of heavy alcohol use enhance the risk of sleeplessness and whether change in and stability of sleeplessness enhance the risk of heavy alcohol use. To examine gender-specific risks, the analyses were stratified by gender. To the best of our knowledge, the directionality of this relationship has not been previously investigated for men and women separately.

## Methods

### Sample and procedure

The current data come from a longitudinal general population health study (the Tromsø Study) conducted in the city of Tromsø in Norway. The main purpose of this cohort study was originally to identify risk factors of cardiovascular disease but has been expanded to include measures of various health- and illness-related factors [17]. The study has been repeated seven times since 1974 and includes extensive physical health examinations in addition to health survey data. To increase the statistical power of the analyses, the present study used survey data only from the fourth (hereafter labeled T1) and sixth (labeled T2) waves, which were conducted in 1994–1995 and 2007–2008, respectively. At T1, 27,158 persons participated, representing a response rate of 72.3% (from all eligible participants aged 25 or older, *N* = 37,558). At T2, those who participated at T1 were invited along with random selections of those aged 30–39 (10%), those aged 43–49 years (40%), as well as all inhabitants aged 40–42 and 60–87. In total, 12,984 (65.7%) participated. Of these, 10,325 (79.5%) also participated at T1. Missing data were handled using multiple imputation, resulting in a final sample of 9941 (men *N* = 4611, women *N* = 5330). Respondents aged ≥70 years were excluded because they completed a different questionnaire without the variables of interest to the present study.

### Measures

*Sleeplessness* at T1 and T2 was measured by asking to what extent the respondent was bothered by sleeplessness (never, 1–3 times per month, once a week, or more than once a week). These categories were dichotomized into no or infrequent sleeplessness (never or 1–3 times per month, hereafter called low score) and experiencing sleeplessness (approximately once a week and more than once a week, hereafter called high score). This variable was used both as an outcome (with two categories, dichotomized as described above) and as a predictor variable. To study change in and stability of sleeplessness as predictors, a variable based on the T1 and T2 responses was created with the following four groups: (0) stable sleeplessness at both T1 and T2 (high score T1, high score T2), (1) increasing sleeplessness (low score T1, high score T2), (2) decreasing sleeplessness (high score T1, low score T2), and (3) stable absence of sleeplessness (low score T1, low score T2). The latter category served as the reference group.

*Alcohol use*. At T1, the respondents reported the number of days in which alcohol was consumed within a normal month (numerical response), as well as the number of units of beer, wine or spirits normally consumed within a two-week period (numerical responses). In addition, a question about the frequency of alcohol consumption corresponding to at least five small bottles of beer, a bottle of wine or a quarter bottle of spirits in one sitting during the past 12 months was included. The response categories ranged from not at all in the past year to three or more times per week. To ensure that the alcohol variables could be combined into a single variable, a principal component analysis of the T1 alcohol variables was conducted, and the results suggested a one-factor solution (*explaining R*^*2*^ = 55.9% of the variance). A summative scale of the four variables was constructed and ranked in ascending order under the assumption that an individual’s ranking in the distribution is of greater importance than the observed scores of the variables. At T2, alcohol consumption and alcohol problems were measured by the Alcohol Use Disorders Identification Test (AUDIT) [18]. To assist comparison of the T1 and T2 sum scores, the following AUDIT items were used: “How often do you drink alcohol” (never to 4 or more times per week), “How many units of alcohol do you usually drink when you drink alcohol?” (1–2 to 10 or more), and “How often do you drink six units of alcohol or more in one occasion?” (never to daily or almost daily). A principal component analysis of the T2 variables also extracted a single eigenvalue > 1 (*R*^*2*^ = 58.3%), supporting the creation of a single composite score. The variables were summed and ranked in ascending order.

The ranked T1 and T2 alcohol-use sum scales were dichotomized. Due to gender differences in alcohol consumption, one variable for women and one variable for men were created. For men, the top 11.7% of the T1 sample and the top 12.4% of the T2 sample were categorized as high consumers. For women, the top 7.1% of the T1 sample and the top 6.9% of the T2 sample were categorized as high consumers. For men, the percentage scoring above the cut-off value is somewhat lower than the 12-month prevalence rates for men with alcohol abuse/dependence in Norway, whereas for women, the percentage scoring above the cut-off corresponds to the prevalence rates for alcohol-use disorders for women in Norway [19]. The distribution of the scores necessitated the chosen cut-off values and explains why different cut-off values were chosen for T1 and T2. Alcohol use was used both as an outcome variable (with two categories) and as a predictor variable (with four categories). When serving as an outcome variable, T2 consumption of alcohol was dichotomized as described above. To use change in and stability of high consumption of alcohol as predictors, a variable combining the T1 and T2 variables was computed similar to the one described for sleeplessness: (0) stable high alcohol consumption at T1 and T2 (high T1, high T2), (1) increasing alcohol consumption (low T1, high T2), (2) decreasing alcohol consumption (high T1, low T2), and (3) stable low alcohol consumption (low T1, low T2). The last category was the reference group in the analyses.

*Mental health* at T1 was measured by the CONOR Mental Health Index. The index consists of seven items measuring positive and negative emotionality and anxiousness and is partly based on the General Health Questionnaire, partly on the Hopkins Symptom Checklist and partly on other mental health measures. The CONOR Mental Health Index has been found to have high internal consistency, low to moderate sensitivity and high specificity in detecting mental distress [20]. A composite score was created.

*Somatic health problems* at T1 were measured by items covering past or present heart attack, angina pectoris, diabetes, cancer, asthma, stroke, epilepsy or fibromyalgia. A dichotomous variable was created that categorized all respondents who had experienced any somatic health problems in one group and the respondents who had not had somatic health problems in another group.

*Social security benefits at T1*. Respondents were asked to indicate if they received the following social security benefits: sick leave benefits, rehabilitation benefits, disability pension, social welfare benefits or unemployment benefits. A dichotomous variable was created: (0) receiving no social benefits and (1) had received any kind of social benefits.

*Body mass index (BMI) at T1.* Weight was reported in kilograms and height in cm. BMI was calculated by dividing weight (kg) by height squared (cm).

*Demographic information*. Education was measured by asking the respondents to indicate the highest level of completed education at T1. The response categories were primary/secondary school, vocational school/started high school, high school diploma, college/university < 4 years or college/university > = 4 years. The respondents stated their marital status, which was categorized as (0) divorced, separated or widower, (1) single and (2) married/registered partnership. Age at the time of participation and gender were also registered at T1.

### Statistical analyses

Hierarchical logistic regression analyses were performed using the statistical software IBM SPSS 24 for Windows. The covariates included in the hierarchical models were chosen based on known relationships to alcohol use or sleeplessness in general [21–28]. In the first model, the change and stability variables for alcohol use or sleeplessness were entered alone to estimate the crude odds ratios. Next, demographic covariates (i.e., age, education and marital status) were added to model 2. In model 3, the dichotomous T1 variable of the outcome variable was entered, and in the fully adjusted model, health and psychosocial covariates were added, i.e., mental distress, somatic health problems, having received social security benefits and BMI. All analyses were stratified by gender, as different criteria were used for men and women to categorize change in and stability of alcohol use.

### Treatment of missing values

Multiple imputation (IBM SPSS 24 for windows) was applied to achieve a more complete dataset. Only persons participating at both T1 and T2 were included in the data to be imputed. The imputation model used all T1 and T2 variables included in the final regression analyses to create five imputed datasets. Missing values were reduced from 2.7% for T1 sleeplessness, 11.7% for T2 sleeplessness, 23.7% for the T1 alcohol frequency sum scores, 15.8% for the T2 alcohol frequency sum scale, and 3.7% for the CONOR mental health index. Complete case analyses were also run, and the differences were only negligible. Hence, only results from the multiply imputed datasets will be shown.

## Results

Descriptive statistics at T1 for the male and female sample of the study are displayed in Table 1.

**Table 1:** Descriptive statistics of the sample

	Men		Women	
	*N*	*%*	*N*	*%*
Sex	4611	46.4%	5330	53.6%
Education T1				
Primary school	1373	29.8%	1935	36.3%
Vocational high school/started high school	1380	29.9%	1488	27.9%
High school diploma	348	7.5%	479	9.0%
College/university < 4 years	841	18.2%	712	13.4%
College/university > 4 years	669	14.5%	716	13.4%
Marital status T1				
Married/registered partner	3047	66.1%	3323	62.3%
Single	1060	23.0%	1037	19.5%
Divorced/separated/widowed	504	10.9%	970	18.2%
Social welfare benefits T1				
Yes	601	13%	1146	21.5%
Change in and stability of alcohol use				
Stable high, high T1-high T2	231	5.0%	126	2.4%
Increasing, low T1-high T2	340	7.4%	242	4.5%
Decreasing, high T1-low T2	308	6.7%	254	4.8%
Stable low, low T1-low T2	3732	80.9%	4708	88.3%
Change in and stability of sleeplessness				
Stable sleeplessness, high T1-high T2	190	4.1%	527	9.9%
Increasing sleeplessness, low T1-high T2	355	7.7%	844	15.8%
Decreasing sleeplessness, high T1-low T2	198	4.3%	283	5.3%
Stable no sleeplessness, low T1-low T2	3868	83.9%	3676	69%
Somatic health problems T1				
Yes	763	16.5%	1240	23.3%
	*M*	*SD*	*M*	*SD*
Age	47.1	11.5	46.7	12.3
Mental distress T1 (range 7–27)	10.2	2.5	10.7	3.1
BMI	25.8	3.2	24.9	4.0

The frequency and percentage are displayed for categorical variables, and the mean (M) and standard deviation (SD) are presented for continuous variables

### Change in and stability of alcohol consumption and sleeplessness at T2

The crude estimates showed that men with a stable high, increasing or decreasing consumption of alcohol had significantly higher odds of reporting sleeplessness at T2 compared with the reference group (see Table 2). In model 3, when adjusting for sleeplessness at T1, the group with decreasing alcohol use was no longer significantly different from the reference group. The effects of stable high and increasing consumption of alcohol were robust as the addition of adjustment variables in three separate blocks did not alter these estimates noticeably. A similar regression analysis for women found no significant relationships (see Table 3).

**Table 2:** Logistic regression analysis of sleeplessness for men according to change in alcohol use

Alcohol use	Model 1^a^		Model 2^b^		Model 3^c^		Model 4^d^	
	*OR*	*CI*_*.95*_	*OR*	*CI*_*.95*_	*OR*	*CI*_*.95*_	*OR*	*CI*_*.95*_
Stable high, high T1-high T2	2.21***	1.57–3.13	2.40***	1.69–3.41	2.10***	1.41–3.12	2.11***	1.43–3.13
Increasing, low T1-high T2	1.69***	1.24–2.30	1.91***	1.39–2.61	1.97***	1.40–2.77	1.94***	1.38–2.73
Decreasing, high T1-low T2	1.42*	1.01–1.99	1.42*	1.01–2.00	1.36	0.94–1.98	1.38	0.95–2.00
Stable low, low T1-low T2	Ref.		Ref.		Ref.		Ref.	

*Notes*. *** *p* < .001, ** *p* < .01, * *p* < .05. *CI*_*.95*_ = 95% confidence interval

^a^Crude estimates

^b^Adjusted for age, education and marital status

^c^Adjusted for Model 2 + sleeplessness at T1

^d^Adjusted for Model 3 + mental health problems, somatic health problems, BMI, and social welfare benefits

**Table 3:** Logistic regression analysis of sleeplessness for women according to change in alcohol use

Alcohol use	Model 1^a^		Model 2^b^		Model 3^c^		Model 4^d^	
	*OR*	*CI*_*.95*_	*OR*	*CI*_*.95*_	*OR*	*CI*_*.95*_	*OR*	*CI*_*.95*_
Stable high, high T1-high T2	0.92	0.56–1.49	1.11	0.67–1.85	1.12	0.65–1.94	1.11	0.64–1.92
Increasing, low T1-high T2	1.02	0.75–1.39	1.31	0.95–1.80	1.24	0.88–1.75	1.28	0.90–1.81
Decreasing, high T1-low T2	1.07	0.79–1.46	1.12	0.82–1.53	1.07	0.76–1.52	1.05	0.74–1.49
Stable low, low T1-low T2	Ref.		Ref.		Ref.		Ref.	

*Notes*. *** *p* < .001, ** *p* < .01, * *p* < .05. *CI*_*.95*_ = 95% confidence interval

^a^Crude estimates

^b^Adjusted for age, education and marital status

^c^Adjusted for Model 2 + sleeplessness at T1

^d^Adjusted for Model 3 + mental health problems, somatic health problems, BMI, and social welfare benefits

### Change in and stability of sleeplessness and high consumption of alcohol at T2

The crude estimates showed that stable and increasing sleeplessness among men predicted higher odds of high consumption of alcohol at follow-up compared with the reference group. Men with decreasing sleeplessness between T1 and T2 did not differ significantly from the reference group. These findings were robust as the addition of adjustment variables did not alter the associations markedly (see Table 4). Again, a comparable analysis for women revealed no significant relationships (see Table 5).

**Table 4:** Logistic regression analysis of alcohol use for men according to change in sleeplessness

Sleeplessness	Model 1^a^		Model 2^b^		Model 3^c^		Model 4^d^	
	*OR*	*CI*_*.95*_	*OR*	*CI*_*.95*_	*OR*	*CI*_*.95*_	*OR*	*CI*_*.95*_
Stable sleeplessness, high T1-high T2	2.01***	1.38–2.91	2.32***	1.58–3.44	1.98***	1.30–3.02	1.84**	1.19–2.85
Increasing, low T1-high T2	1.80***	1.34–2.40	1.96***	1.45–2.65	1.81***	1.31–2.51	1.78***	1.28–2.47
Decreasing, high T1-low T2	1.10	0.71–1.70	1.08	0.69–1.68	1.02	0.63–1.65	1.00	0.59–1.58
Stable no sleeplessness, low T1-low T2	Ref.		Ref.		Ref.		Ref.	

*Notes*. *** *p* < .001, ** *p* < .01, *p < .05. *CI*_*.95*_ = 95% confidence interval

^a^Crude estimates

^b^Adjusted for age, education and marital status

^c^Adjusted for Model 2 + symptoms of alcohol use at T1

^d^Adjusted for Model 3 + mental health problems, somatic health problems, BMI, and social welfare benefits

**Table 5:** Logistic regression analysis of alcohol use for women according to change in sleeplessness

Sleeplessness	Model 1^a^		Model 2^b^		Model 3^c^		Model 4^d^	
	*OR*	*CI*_*.95*_	*OR*	*CI*_*.95*_	*OR*	*CI*_*.95*_	*OR*	*CI*_*.95*_
Stable sleeplessness, high T1-high T2	0.86	0.56–1.30	1.25	0.80–1.94	1.20	0.80–1.94	1.29	0.79–2.11
Increasing, low T1-high T2	1.11	0.82–1.50	1.31	0.97–1.78	1.30	0.97–1.79	1.34	0.97–1.85
Decreasing, high T1-low T2	1.00	0.59–1.58	1.16	0.70–1.91	1.22	0.72–2.06	1.27	0.74–2.17
Stable no sleeplessness, low T1-low T2	Ref.		Ref.		Ref.		Ref.	

*Notes*. *** *p* < .001, ** *p* < .01. *p < .05. *CI*_*.95*_ = 95% confidence interval

^a^Crude estimates

^b^Adjusted for age, education and marital status

^c^Adjusted for Model 2 + symptoms of alcohol use at T1

^d^Adjusted for Model 3 + mental health problems, somatic health problems, BMI, and social welfare benefits

## Discussion

In the present study, we observed a bidirectional longitudinal relationship between high consumption of alcohol and sleeplessness; that is, stable high and increasing high consumption of alcohol predicted a higher risk of sleeplessness, and stable and increasing sleeplessness predicted a higher risk of high consumption of alcohol. This bidirectionality was only evident among men.

The risk of experiencing sleeplessness was slightly higher for men with stable high consumption of alcohol compared to men with increasing consumption of alcohol. However, it is not possible to discern if the risk for those with stable high consumption was caused by their current heavy drinking as measured at T2 or by a more severe and chronic condition represented by stable high consumption of alcohol. The analyses also showed that men with decreasing consumption of alcohol between T1 and T2 did not have a higher risk of sleeplessness at T2 compared with the reference group, after adjusting for sleeplessness at T1, which indicates that reducing alcohol use may improve sleep and lower the risk of sleeplessness in the future, corroborating the findings of the study by Crum et al. [14]. The same pattern was found in the analyses of how change in and stability of sleeplessness among men were related to high T2 alcohol consumption. The risk of high consumption of alcohol at T2 was slightly higher for men with stable sleeplessness over time compared with men experiencing increasing sleeplessness between T1 and T2. Again, decreasing sleeplessness between T1 and T2 was not related to higher risk of heavy alcohol use at T2 compared with the reference group. This indicates that relieving sleeplessness may reduce the risk of heavy drinking over time. In addition, the results showed that the predictive effects of stable high and increasing consumption of alcohol were approximately of the same magnitude as the effects of stable and increasing sleeplessness. Together, these results reasonably suggest a reciprocal relationship between sleeplessness and high consumption of alcohol for men.

The existing literature has shown in a relatively consistent manner that experiencing insomnia enhances the risk of heavy alcohol use (e.g. [6–8]). One explanation may be that the sedating effects of alcohol may cause people struggling with insomnia to use alcohol as a hypnotic to a greater degree than others [29]. Another explanation may be observed changes in sleep architecture (i.e., longer duration of deep-level sleep during the first half of the night) and improved mood after alcohol exposure among people struggling with insomnia relative to healthy controls [30]. However, such favorable sleep effects are short-lived. Tolerance to the positive effects of alcohol on sleep develops within three days [3], and as such continued use of alcohol as a hypnotic causes further sleep disruption both acutely and after discontinuation of use [31], ultimately exacerbating the insomnia symptoms [32]. With the negative effects of alcohol on sleep in mind, it is somewhat surprising that the majority of studies investigating alcohol use as a predictor of insomnia do not find a significant effect. Our results do not substantiate this tendency though, as stable high and increasing consumption of alcohol were significantly related to a higher risk of insomnia at T2 for men. Several of the previously published non-significant results were based on the same random sample, the Penn State Sleep Cohort [9–11], and the possibility that sample-specific issues may have impacted the results cannot be excluded. However, other studies have also failed to establish heavy alcohol use as a significant predictor of insomnia over time [12, 13]. The sample sizes of these studies were small to moderate (from 150 to 1395), thus reducing the precision of the estimates. Our results are more in line with the results of the study by Crum et al. [14], who found that individuals with persistent alcohol dependence had greater odds of insomnia 13 years later. Despite the large sample size in our case, which strongly substantiates our findings, future replication in a large population-based sample is recommended.

To a certain degree, our results corroborate the findings of Haario et al. [15]. Interestingly, Haario and colleagues [15] did not find a significant gender difference. Their sample consisted of more than 80% women, whereas our sample was more equally balanced in terms of gender (53.7% women). The gender-specific cut-off values that we used make conclusions about gender as a significant moderating variable uncertain, although the stratified results certainly point in that direction. Other cross-sectional studies have found gender differences, in line with the results of our study. Jaussent et al. [33] found that alcohol negatively predicted insomnia symptoms among women in a study of community-dwelling elderly. For men, alcohol use was an insignificant predictor of insomnia symptoms. Peretti-Watel and colleagues [34] found drug use for insomnia to be related to alcohol use only among men. In line with our results, one may speculate about whether men struggling with insomnia self-medicate with alcohol to fall asleep to a greater degree than women. However, as our results for women differ than those reported by Haario et al. [15], gender differences in the relationship between alcohol use and insomnia symptoms need to be further explored in more large-scale, prospective studies, preferably also including measures of motives for drinking alcohol.

### Strengths and limitations

A major strength of this study is the generalizability provided by the large sample size at both measurement points, as well as the balanced representation of women and men. Moreover, the precision of the estimates was improved by the inclusion of several covariates. Although observational in nature and thus prohibiting causal inferences, the T1-T2 change and stability variables make the present study informative about possible causal relations between sleeplessness and heavy alcohol use. However, it is important to emphasize that this study is observational, and as such, it is not possible to draw conclusions regarding causality based on our study.

There are some limitations in the present study that should be mentioned. First, the study is based on self-report data, and sleeplessness was measured by a single item, which may constrict the validity of this sleep measure. The main diagnostic criteria for insomnia are, according to ICD-10, difficulty with initiating or maintaining sleep, as well as early awakening. Moreover, dissatisfaction with sleep quality and experience of daytime impairment need to be present [35]. Our general question about sleeplessness thus missed several specific aspects of insomnia as a sleep disorder; however, sleeplessness represents a core functional symptom of insomnia that covers both difficulties initiating and maintaining sleep. We therefore consider sleeplessness a valid and clinically relevant indicator of sleep problems and believe that the results of our study are comparable with those of other studies investigating sleep problems related to insomnia. Alcohol use was measured using two slightly different questions at T1 and T2, which may increase the measurement variance between these two occasions. We attempted to account for this limitation by using ordinal ranks rather than the original scores of the variables. The low mean level of alcohol consumption (results not shown) aligns with a tendency to underreport alcohol use in population-based studies [36]. It is thus possible that the persons drinking the most did not participate in the study. In another general population-based Norwegian health survey [37], alcohol use only moderately affected drop-out, which suggests that attrition between T1 and T2 due to drinking is unlikely to have greatly affected the sample. The general selection bias arising from people with mental disorders abstaining from participating in population studies [38] is less relevant because the Tromsø Study was launched as a general health survey and not as a study of alcohol use or sleep problems.

## Conclusion

While the results of the present study clearly show a bidirectional relationship between alcohol use and sleeplessness among men, more research is needed to understand possible gender-specific causal mechanisms. A clinical implication of the present study is that for prevention and treatment purposes, health professionals should include questions about alcohol use for men who present with sleep problems and vice versa.

## Abbreviations

AUDIT: Alcohol Use Disorder Identification Test
BMI: Body Mass Index
